# Prevalence of Modic changes in the lumbar vertebrae and their associations with workload, smoking and weight in northern China

**DOI:** 10.1038/srep46341

**Published:** 2017-04-12

**Authors:** Chao Han, Ming-jie Kuang, Jian-xiong Ma, Xin-long Ma

**Affiliations:** 1 Department of Orthopedics, Tianjin Hospital, No. 406 Jiefang South Road, Hexi District, Tianjin 300211, P.R. China; 2 Tianjin Medical University, Tianjin 300070, P.R. China

## Abstract

The distribution of Modic changes (MCs) in the lumbar endplates and the evaluation of the relationships between MCs and risk factors are vital for research into MCs. The T1-weighted and T2-weighted sagittal MRI scans of 210 patients who exhibited lumbar intervertebral disc degeneration were retrospectively reviewed. The patients’ weights, genders, smoking statuses, physical activity levels and specific types of MC were recorded. The associations between MCs and risk factors, such as physical work, smoking and body mass index, were also analysed. MCs were observed in 47 patients (22.4%), including 16 males and 31 females. Among all patients, the L5/S1 lumbar level was most likely to suffer MCs. The MCs were predominantly type II. MCs occurred more often in obese patients than in normal and overweight patients (P < 0.05). Patients whose jobs required heavy labour were more likely to develop MCs (P < 0.05). Heavy work and obesity were related to type III MCs more strongly than the other types (P > 0.05). Smoking seemed not to be correlated with the incidence of MCs (P > 0.05). Gender, obesity and heavy work were strongly associated with MCs. Biomechanical factors may play a critical role in the development of MCs.

Modic changes (MCs) are lesions of the vertebral endplate and adjacent bone marrow that are visible on magnetic resonance imaging (MRI). MCs are notably common in people who have the relevant symptoms^1^. The importance of MCs has been highlighted in many studies due to their association with low back pain (LBP)^2^,^3^. Three different types of subchondral signal abnormalities in the vertebral body marrow were first independently described by de Roos *et al*.^4^ and Modic *et al*.^5^. Modic type I changes are reflected by a hypointense signal on T1-weighted [T1W] and a hyperintense signal on T2-weighted [T2W] sequences. Fissured endplates with adjacent vascular granulation tissue within the bone marrow are found in such lesions. Modic type II changes appear as hyperintense signals on both T1W and T2W sequences. Disruption of the endplates as well as histological fatty replacement of the adjacent bone marrow can be detected in this type of lesion. Modic type III changes elicit hypointense signals on T1W and T2W sequences. Lesions with sclerosis corresponding to the endplate are observed^6^. MCs have been considered pathological spinal conditions that are closely related to many degenerative diseases of the spine^7^,^8^,^9^.

In reviews of the currently available evidence, some studies have already focused on the detailed knowledge of the aetiologies of and risk factors for MCs. Albert *et al*. hypothesized that MCs are possibly induced by intervertebral disc herniation^8^. Hu *et al*. ascribed MCs to severe intervertebral disc degeneration^10^. Crock proposed the concept of “internal disc disruption” in which repeated trauma to the disc can result in micro-fissures on the endplate. Genetic factors have been proven that have the ability to affect the severity and development of disc degeneration, which suggests that such factors may influence the distributions of MCs in different races^11^. Because Modic type II changes are histologically characterized by fatty replacement^5^, it is possible that this type of pathological change is strongly linked to obesity. Previous studies have reported that overweight plays a key role in the cause of disc degeneration and that it may also be associated with Modic changes^12^. Some authors have hypothesised that smoking may result in reduced bone mineral content due to the contraction of small arteries in the discs and thus increase the likelihood of developing micro-fractures^13^,^14^. Heavy labour may increase the burden on the vertebral discs and weaken the bone structure, which would eventually enhance the possibility of developing MCs^15^.

Although there are many studies regarding the clinical aspects of MCs^16^,^17^,^18^,^19^,^20^,^21^,^22^,^23^,^24^, appropriate epidemiological investigations that reveal the relationship between MCs and risk factors, such as physical work, smoking and body mass index, for specific races are rare. A systematic study of MCs in the lumbar region would provide fundamental epidemiological insights into MCs in addition to important indicators for clinical observation and research. It is very important to provide an accurate assessment of the classification and distribution of MCs especially in terms of the relationships to the risk factors. We performed this retrospective study to evaluate the general distribution of MCs in the Chinese population to provide an improved understanding of the contributions of these risk factors to the progression of MCs

## Materials and Methods

### Study population

Patients with low back pain in Tianjin Hospital between February 2014 and October 2015 were examined in the present study. MRI and X-ray imaging were applied to all patients to evaluate the presence of MCs. To remove interference, patients with tuberculosis, tumours, infections and trauma were excluded. The study was conducted in accordance with the *Declaration of Helsinki* and was approved by the Ethics Committees of Tianjin Hospital. Written informed consent was obtained from all patients.

### MRI assessments of the MCs

#### Magnetic resonance imaging (MRI)

MRIs were performed with a Siemens Avanto 1.5-T Magnetom (Signa EXCITE HD 1.5 T), and a body spine surface coil was used with the patient in the supine position. The imaging protocol consisted of sagittal and axial T1- and T2-weighted sequences. Sagittal T1-weighted turbo spin echo (TR/TE 420/13.6 ms) and sagittal T2-weighted turbo spin echo (TR/TE 3000/100 ms) sequences with a layer thickness/layer spacing of 4 mm/1 mm, a 384 × 160 matrix, and a FOV of 28 × 28/W were collected twice.

The MRIs of all lumbar vertebral discs (L1 to S1, both the left and right sides) were independently read in random order by one radiologist with experience in musculoskeletal MRIs and one orthopaedist. The readers were blinded to the clinical data, and they decided whether MCs were present and determined the types of MCs. The eFilm version 3.4 (Merge Healthcare, Hartland, Wisconsin) imaging reading software was used. Disagreements between the reviewers were settled by discussion, and if no consensus could be reached, a third reviewer made the final decision as an adjudicator.

#### Types of MCs

**Figure 1:**
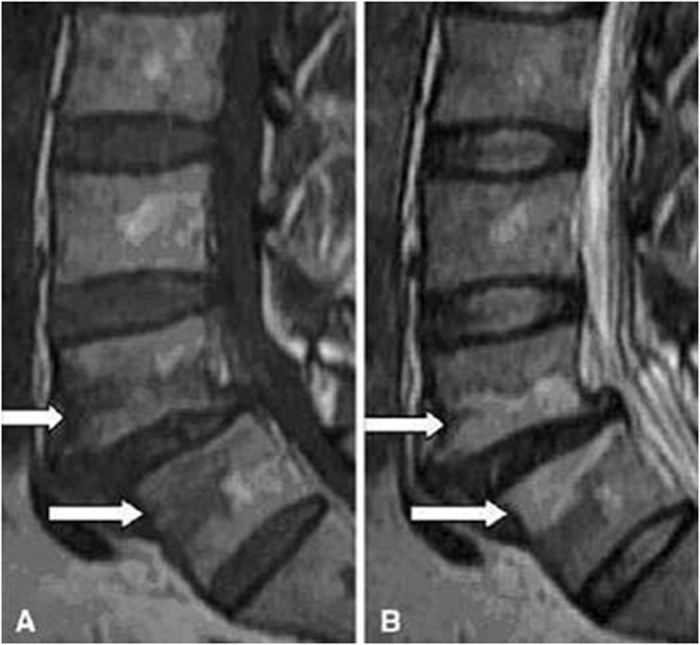
Magnetic resonance images of type I MCs at the L4–5 level in the lumbar spine.

**Figure 2:**
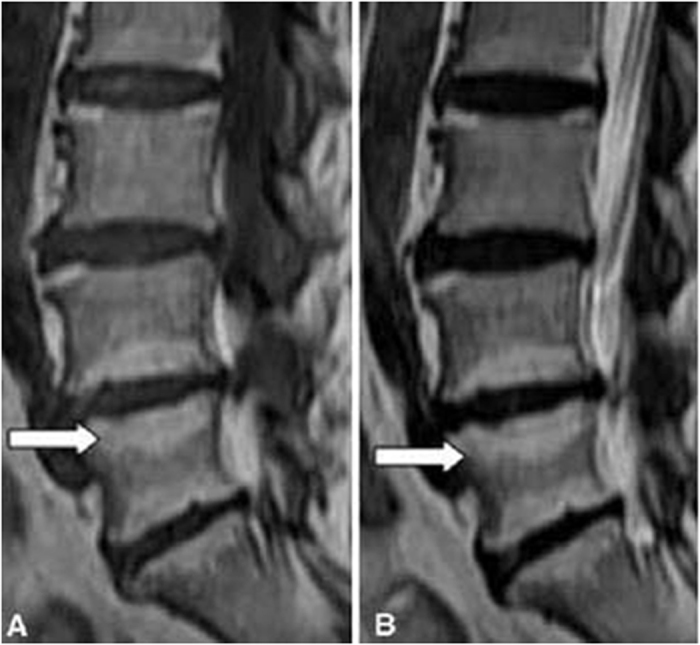
Magnetic resonance images of type II MCs at the L4–5 and L5-S1 levels in the lumbar spine.

**Figure 3:**
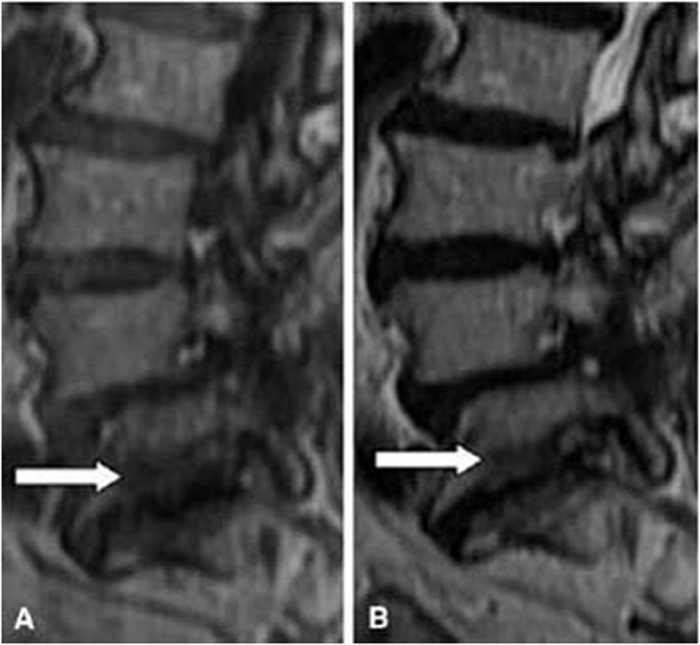
Magnetic resonance images of type III MCs at the L5-S1 level in the lumbar spine.

According to definitions of Modic *et al*.^4^,^5^, the MCs was classified as type I (presenting low signal intensity on T1-weighted images and high signal intensity on T2-weighted images; these lesions consist of bone marrow oedema and micro-fractures; Fig. 1), type II (high signal intensities on T1-weighted images and isointense or slightly hyperintense signals on T2-weighted images; these lesions consist of fatty replacements of the red bone marrow or bone marrow necrosis; Fig. 2), or type III (low signal intensities on both T1- and T2-weighted images; these lesions consist of sclerotic bone replacement of the bone marrow fat deposition; Fig. 3).

#### Assessment criteria

The patients’ weights were assessed by the BMI for Asians^25^ (BMI = weight in kilograms/height in meters squared). The patients were divided into the following groups based on BMI: 18.5 ≤ BMI < 22.9 kg/m^2^ (normal weight), 23 ≤ BMI < 24.9 kg/m^2^ (overweight), and BMI ≥ 25 kg/m^2^ (obese).

The amount of work was divided into light physical work (mainly sitting), moderate physical work (sitting/walking), and hard physical work (heavy working) according to the method of Leboeuf^15^ Moreover, the patients’ feelings regarding their amount of physical work were also take into account for calibration. The group assignments could be changed if a patient believed that he/she could not totally bear the current labour intensity.

The patients were divided into non-smoking, mild smoking (1–19 cigarettes/day), and heavy smoking ( ≥ 20 cigarettes/day) groups based on their smoking habits according the method of Jensen^7^.

### Data analysis

The statistical analyses were performed with the Statistical Package for the Social Sciences (SPSS), version 16.0. The concordance between the two raters was examined using the Kappa concordance test. Theχ^2^ test (or Fisher’s exact test when counts below 5 were expected) was used to compare the categorical variables between the groups. A multivariate logistic regression model was developed to assess the variables that were associated with an increased risk of presenting with MCs. P-values below 0.05 were regarded as statistically significant.

## Results

### Clinical characteristics of the MCs

**Figure 4:**
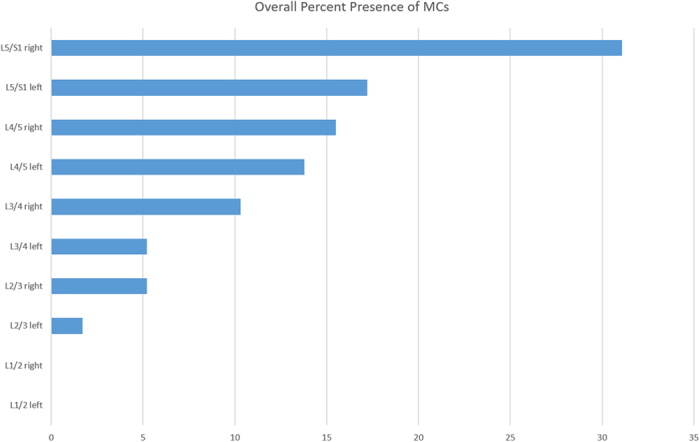
The overall prevalences of MCs according to lumbar disc level in both sides.

Two hundred thirty patients were screened for inclusion. Twelve patients had undergone spinal operations, and 5 had inflammatory spondyloarthropathies. The remaining 213 patients were invited to participate in the study, and 3 refused for of personal reasons (there were no differences between those who refused and those who participated). Therefore, 210 patients (male: 99 and female: 111) were ultimately included. Among the 1100 discs of the 210 patients, a total 58 intervertebral discs of 47 patients exhibited MCs (22.4%). The age range was 40 to 60 years. The distribution of the types of MCs was as follows: type I, 16 (7.6%); type II, 25 (11.9%); and type III, 6 (2.9%). The majority of the MCs occurred at the lowest two lumbar levels (77.6%) with the L5/S1 level on the right side (30.1%) being the most commonly affected (Fig. 4).

### Relationships between MCs and gender

Across all patients (male: 99 and female: 111), the incidence of MCs was significantly higher among the females than the males (27.9% vs 16.2%, P < 0.05; Table 1).

### Relationship between MCs and weight

The incidences of MCs were 17.3% in the normal weight group (n = 98), 17.5% in the overweight group (n = 80), and 50% in the obese group (n = 32). The incidence in the obese group was significantly greater than those in the normal weight and overweight groups (P < 0.05). However, no significant difference was found between the normal weight groups and the overweight group (P > 0.05; Table 2).

### Relationships between MCs and physical work

Significant differences were found in the incidence of MCs according to the level of physical work. Among all patients, 14.8% were categorised into the light physical work group (n = 54), 16.3% were categorised into the moderate physical work group (n = 99), and 40.4% were categorised into the hard physical work group (n = 57) (*P* < 0.05; Table 3).

### Relationship between MCs and smoking

No significant differences in the incidences of MCs were found according to smoking status. Among all patients, 25.4% were non-smokers (n = 138), 11.3% were mild smokers (n = 53), and 31.6% were heavy smokers (n = 19; Table 4).

### Relationships of the types of MCs with the influencing factors

#### Gender

Across all study participants, the patients with Type I MCs included 6 males and 10 females (37.5% and 32.3%, respectively), patients with Type II MCs included 8 males and 17 females (50% and 54.8%, respectively), and the patients with Type III MCs included 2 males and 4 females (12.5% and 12.9%, respectively). No significant differences were found between the males and females in any of the types of MCs (P > 0.05; Table 5).

#### Weight

There were no significant differences among the patients with Types I and II MCs (P > 0.05). However, the pooled data revealed that the obese patients were more vulnerable to Type III MCs (P < 0.05; Table 5).

#### Work

This pattern associated with work level was consistent with that of weight; i.e., no significant differences were found between the patients with Type I and Type II MCs (P > 0.05), however, compared with the moderate physical work group, the patients in the hard physical work group were more likely to exhibit Type III MCs (P < 0.05; Table 5).

#### Multivariate logistic regression

In the multivariate logistic regression analyses of all confounding factors (i.e., heavy labour, obesity, and female gender), the incidence of an MC was significantly and positively associated with heavy labour (OR: 11.431; 95% CI: 6.754–19.349; P < 0.0001), obesity (OR: 1.856; 95% CI: 1.189–2.899; P = 0.0065) and the female gender (OR: 1.609; 95% CI: 1.031–2.510; P = 0.0362). Heavy labour was most strongly correlated with the presence of an MC (Table 6).

## Discussion

MCs are signal changes in the vertebral bone marrow and adjacent endplates on MRI. MCs were first reported by Roos in 1987^4^, and Modic subsequently systematically described MCs in 1988^5^. In our study, we investigated the distribution of MCs in lumbar endplates and evaluated the relationships of MCs with some risk factors. A multivariate logistic regression analysis that included the genders, weights, and work intensities of patients with MCs was also applied to identify the “culprit” that was responsible for MCs among these risk factors. Our study confirmed that physical work was the factor that was most responsible for MCs, and this finding accords with those of previous reports^7^,^15^.

Previous research has demonstrated that many factors may affect the prevalence of MCs, e.g., long-term hard physical work may overstrain the vertebral bodies, aggravate the progression of disc degeneration and weaken the bone structure. This process could result in micro-fractures and fissures in the endplates^15^. Long-term smoking can not only increase the intra-abdominal pressure and alter hormone levels but also has the abilities to slow blood flow in the discs, diminish the mineral content of the bone and ultimately affect the biological repair functions of the vertebral bodies^14^. Overweight is also a substantial risk factor for MCs. Obese patients are more vulnerable to fatigue fractures of the vertebrae^7^. In our study, the incidence of MCs in patients who performed hard physical work was four times greater than that among those who performed light physical work, and obese patients were more likely to suffer MCs. Osteoporosis could be taken into account and may explain why the females exhibited a higher incidence of MCs than the males. Although there were no statistical differences in the prevalences of MCs according to smoking status, because smoking can induce the contraction of small arteries and affect the blood supply of the vertebral bodies, smoking may play an important role in the delayed healing of micro-fractures. The analysis of the factors related to the types of MCs clearly revealed that the type III MCs were closely associated with physical work and weight.

Mechanical load may exert an important role in the development of MCs^26^. Two possible pathogenic mechanisms might explain this influence. On the one hand, when intervertebral discs are under pressure, the rate of matrix synthesis decreases, and proteoglycan content also decreases. These changes could gradually lower the load-bearing capacity. On the other hand, when degeneration or herniation occurs, shear forces on the endplates may increase due to the loss of the nucleus pulposus. Moreover, changes in the internal material of the disc would also lead to disc-vertebrae dynamic interface suffering greater larger axial and torsional stresses. This process could result in the formation of micro-fractures in the endplate. A previous study reported that the both hard physical work and overweight status can results in a greater load on the endplate, and in conditions of repeated and chronic mechanical loads, the endplate (which is only 0.6 mm thick on average) is easily damaged^27^. As few as 100 repeated applications of 50% to 80% of the ultimate tensile strength to the endplate may result in micro-fractures^28^. Dieën *et al*.^29^ reported that when the load exceeds the limit, the endplate and trabecular bone will collapse, and the degree of collapse depends on the cross-sectional bone mineral density in the vertebra. Karchevsky^30^ also confirmed that micro-fractures and other types of injury are linked to patient weight.

Following micro-fracture, the nucleus pulposus may enter the upper and lower endplate through the micro-fracture and contact the circulatory system. As the largest non-vascular tissue, the nucleus pulposus does not have the opportunity to contact the immune system after the formation of the embryo. Thus, the nucleus pulposus will interact with T and B lymph cells once it contacts the blood circulation. Once the nucleus pulposus enters into the vascular tissue, the immune system might recognize it as a foreign body, which could induce an immunological reaction in the future. Subsequently, crosstalk between the immune system and the bone may occur, which could lead to bone loss due to the dysregulation of T-lymphocyte function and enhance bone absorption activity, which in turn could induce vertebral signal changes^31^. Haemorrhage, oedema, and vascular changes are the typical changes observed in type I MCs, and these changes also reflect the inflammation repair process in endplates and vertebrae following micro-fractures. Burke *et al*.^32^ compared the production levels interleukin-6 (IL-6), interleukin-8 (IL-8) and prostaglandin E2 (PGE2) in disc tissues from patients with different types of MCs and found that the incidence of type I MCs is significantly higher than that of type II MCs. Karppinen *et al*.^33^ reported that IL-1 cluster polymorphisms are significantly associated with MCs. Repeated mechanical loads could accelerate disc degeneration and the formation of micro-fractures. Micro-fractures further encourage the nucleus pulposus to contact the circulatory system, induce autoimmune reactions, and cause MCs. This process may be the real reason that BMI and physical work were positively related to MCs in this study.

This study strengthens the notions that obesity and hard physical work increase the probability of MCs. Approximately 1/3 of the patients with MCs reported that they were required to engage in substantial physical work. Furthermore, obesity was not significantly correlated with MCs when compared with hard physical work. This finding could be also explained by mechanical points, i.e., because obesity is a gradual process, the body can enhance bone strength accordingly and thus easily acclimatize. In contrast, physical work is an external, sudden event that usually involves the application of compression stress and shear forces to the endplates within a very short time, and such stress and force may exceed the capacity of an endplate and potentially result in damage.

Recent systematic reviews have reported the characteristics and locations of MCs in the lumbar spine and to some extent agree with the results of our study. Huang *et al*.^34^ investigated thirty-one studies and found that MCs most commonly occurred in the lowest two levels, and especially in L5/S1, a correlation between MCs and biomechanics was present. Jensen *et al*.^35^ demonstrated MCs are a common finding in patients with non-specific LBP and are associated with pain. Brinjikji *et al*.^36^ demonstrated that MR imaging evidence of Type I MCs is more prevalent in adults of 50 years of age with back pain than in the asymptomatic population. Many factors might affect the development of MCs, and additional studies from multiple perspectives are necessary.

Finally, there are several limitations of our study. First, the sample of patients was relatively small; this was only a retrospective study conducted in one hospital over one year. Second, the treatments of the included patients varied; unfortunately, we did not apply the analyses to the prognoses. Thus, randomized controlled trials or prospective studies with large populations of patients are needed in the future to confirm our results and to evaluate therapies for patients with MCs.

## Conclusions

The female gender, obesity and heavy work were strongly associated with MCs. Biomechanical factors may play a critical role in the development of MCs.

## Additional Information

**How to cite this article**: Han, C. *et al*. Prevalence of Modic changes in the lumbar vertebrae and their associations with workload, smoking and weight in northern China. *Sci. Rep.* **7**, 46341; doi: 10.1038/srep46341 (2017).

**Publisher's note:** Springer Nature remains neutral with regard to jurisdictional claims in published maps and institutional affiliations.

## Figures and Tables

**Table 1:** Associations of gender and MCs.

Gender	MCs, n (%)		Total
	yes	no	
Male	16(16.2%)	83(83.8%)	99
Female	31^①^(27.9%)	80 (72.1%)	111
Total	47(22.4%)	163(77.6%)	210

① The women were more likely to have MCs.

**Table 2:** Distribution of MCs according to weight.

Weight	MCs, n (%)		Total
	yes	no	
Normal weight	17 (17.3%)	81 (82.7%)	98
Overweight	14 (17.5%)	66 (82.5%)	80
Obesity	16^①②^ (50%)	16 (50%)	32
Total	47 (22.4%)	163 (77.6%)	210

① Compared with the normal weight group, P < 0.05;

② compared with the overweight group, P < 0.05.

**Table 3:** Distribution of MCs according to physical work level.

Work	MCs, n (%)		Total
	yes	no	
Light physical	8 (14.8%)	46 (85.2%)	54
Moderate physical	16 (16.2%)	83 (83.8%)	99
Hard physical	23^①②^(40.4%)	34 (59.6%)	57
Total	47 (22.4%)	163 (77.6%)	210

① Compared with the light physical work group, P < 0.05,

② compared with the moderate physical work group, P < 0.05.

**Table 4:** Distribution of MCs according to smoking status.

Smoking	MCs, n (%)		Total
	yes	no	
Non-smoking	35 (25.4%)	103 (74.6%)	138
Mild smoking	6^②^(11.3%)	47 (88.7%)	53
Heavy smoking	6^①②^(31.6%)	13 (68.4%)	19
Total	47 (22.4%)	163 (77.6%)	210

① Compared with the non-smoking group, P > 0.05,

②compared with the mild smoking group, P > 0.05.

**Table 5:** Multivariate analysis of the demographic and clinical characteristics that were associated with MCs.

Type	Gender		Weight			Work		
	Male	Female	Normal weight	Over- weight	Obese	Light physical	Moderate physical	Hard physical
Modic I	6	10	7	6	3	2	6	8
Modic II	8	17	10	5	10	6	10	9
Modic III	2	4	0	3	3^①^	0	0	6^②^
Total	16	31	17	14	16	8	16	23

① The obese patients were more vulnerable to Type III MCs (P < 0.05).

② The patients in the hard physical work group were also more prone to Type III MCs.

**Table 6:** Multivariate logistic regression analysis of the factors associated with the presence of MCs.

Variable	β	SE	Wald χ^2^	P	OR (95% CI)
Heavy labour	2.4364	0.2685	82.3259	<0.0001	11.431 (6.754–19.349)
Obesity	0.6187	0.2273	7.4058	0.0065	1.856 (1.189–2.899)
Female	0.4754	0.2269	4.3887	0.0362	1.609 (1.031–2.510)
Constant	−1.0717	0.2168	24.4395	<0.0001	

OR, odds ratio; CI, confidence interval;
